# Subepithelial rectal gastrointestinal stromal tumor – the use of endoscopic ultrasound-guided fine needle aspiration to establish a definitive cytological diagnosis: a case report

**DOI:** 10.1186/s13256-017-1205-7

**Published:** 2017-03-05

**Authors:** Vitor Ottoboni Brunaldi, Martin Coronel, Danielle Azevedo Chacon, Eduardo Turiani Hourneaux De Moura, Sérgio E. Matuguma, Eduardo Guimarães Hourneaux De Moura, Diogo Turiani Hourneaux De Moura

**Affiliations:** 1 Gastrointestinal Endoscopy Unit, Hospital das Clínicas da Faculdade de Medicina da Universidade de São Paulo, Av. Dr Enéas de Carvalho Aguiar, 225, 6° andar, bloco 3, Cerqueira Cezar, 05403-010 São Paulo, SP Brazil; 2 Patology Unit, Hospital das Clínicas da Faculdade de Medicina da Universidade de São Paulo, Av. Dr Enéas de Carvalho Aguiar, 225, − Andar, bloco –, Cerqueira Cezar, 05403-010 São Paulo, SP Brazil

**Keywords:** Gastrointestinal stromal tumors, Rectum, Endoscopic ultrasound, Immunohistochemistry, Endoscopic ultrasound-fine needle aspiration, Case report

## Abstract

**Background:**

Gastrointestinal stromal tumors are the most common mesenchymal neoplasms affecting the gastrointestinal tract. The stomach is the most common location to be affected, and the rectum one of the rarest, but the whole gastrointestinal tract remains susceptible. Gastrointestinal stromal tumors account for only 0.1% of rectal tumors. Currently, endoscopic ultrasound plays an essential role in the diagnostic process of gastrointestinal stromal tumors, especially when the affected sites have a worse outcome and higher morbidity rates.

**Case presentation:**

We describe the case of a 68-year-old white Japanese man with a history of long-term mild rectal pain and tenesmus. A digital rectal examination revealed a right palpable solid mass ranging from 3 to 7 cm from his anal verge. A colonoscopy was performed and showed a 5 cm elevated lesion covered by normal mucosa, located 4 cm above the pectineal line. Endoscopic ultrasound confirmed the diagnosis of a homogeneous hypoechoic mass with areas of necrosis as a rectal subepithelial lesion originating at the fourth layer (muscularis propria). He then underwent endoscopic ultrasound-guided fine needle aspiration of the lesion, followed by cytological and immunohistochemistry evaluation. The evaluation showed spindle and epithelioid cells of variable sizes, in fascicles separated by stroma, which reacted firmly and consistently to CD117/c-kit and CD34, and negative to desmin and S-100 protein. There was weak staining for nuclear Ki-67 in the tumor cells. A diagnosis of rectal gastrointestinal stromal tumor was confirmed. After a multidisciplinary meeting, an abdominoperineal resection of his rectum was performed. The pathology of the specimen confirmed the diagnosis of rectal gastrointestinal stromal tumor. He is now asymptomatic after 3 months’ follow-up and is on adjuvant therapy with a tyrosine-kinase inhibitor.

**Conclusions:**

Gastrointestinal stromal tumors are rare tumors, and among the variety of primary location sites, the rectum is one of the rarest. The localization of this type of tumor has worse outcomes and higher morbidity rates. We report this rare case to emphasize the need for precise diagnosis and the important role of endoscopic ultrasound-guided fine needle aspiration in such situations.

## Background

Gastrointestinal stromal tumors (GISTs) are the most common mesenchymal neoplasm affecting the gastrointestinal tract [1]. Along with other mesenchymal malignancies, such as leiomyomas, leiomyosarcomas, schwannomas, and lipomas, they present as subepithelial tumors (SETs). For this reason, endoscopic evaluation with standard biopsy does not obtain sufficient tissue for a definitive diagnosis, as it provides only mucosal tissue sampling [2, 3]. Histological diagnosis is essential because treatment and prognosis vary widely among the pathologies mentioned above.

Endoscopic ultrasound (EUS) is an important diagnostic tool for managing GISTs and other SETs. This method provides echographic features that suggest a precise diagnosis, as well as features that may be associated with malignancy [4, 5]. Furthermore, an EUS-guided biopsy is the preferred technique for tissue acquisition of SETs and for definitive morphological diagnosis [6].

This case report illustrates an interesting clinical scenario of a GIST which has been identified in an unusual location, in which EUS plays a key role in diagnosis.

## Case presentation

This is the case of a 68-year-old white Japanese man with a history of long-term mild rectal pain and tenesmus for the last year, denying lower gastrointestinal bleeding and weight loss. Hypertension is his only comorbidity; it is treated with losartan 50 mg administered orally daily. His father died of acute myocardial infarction and his mother died of metastatic gastric cancer. He denied alcohol abuse and any drug addiction including tobacco. Concerning his history of surgery, he had an uncomplicated laparoscopic cholecystectomy due to gallstones 10 years ago. At the first medical consultation, he presented awake and alert, appeared healthy, and looked his stated age. His vital signs were within normal limits, with a blood pressure of 130×70 mmHg and a heart rate of 80 beats per minute (bpm) assessed by radial pulse palpation. A head and neck examination were also normal. An examination of his lungs revealed normal resonant percussion and auscultation was clear. Heart auscultation showed S1 heard best at apex with normal intensity and S2 heard best at base, normal splitting, without any extra sounds. Observation of his abdomen evinced four small laparoscopic scars and auscultation found slightly hyperactive bowel sounds. Abdominal palpation found neither tenderness nor masses. Regarding proctologic examination, an inspection of his anus found no lesion. A digital rectal examination revealed normal anal sphincter tone but showed a right palpable solid mass ranging from 3 to 7 cm from his anal verge. There was no blood on stool and his bulbocavernosus reflex was preserved.

**Fig. 1:**
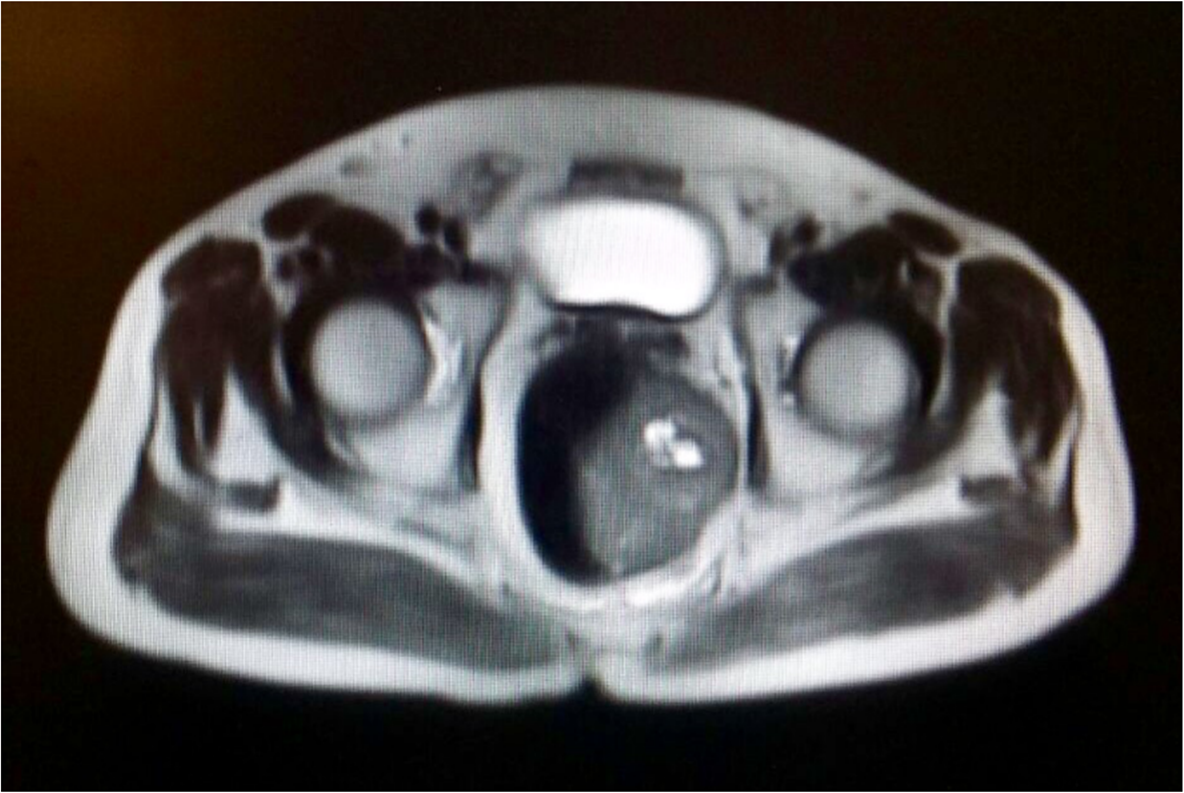
Axial T1-weighted magnetic resonance image shows a well-defined round mass, with low signal intensity on T1-weighted image and on T2-weighted image, arising from the distal rectum

**Fig. 2:**
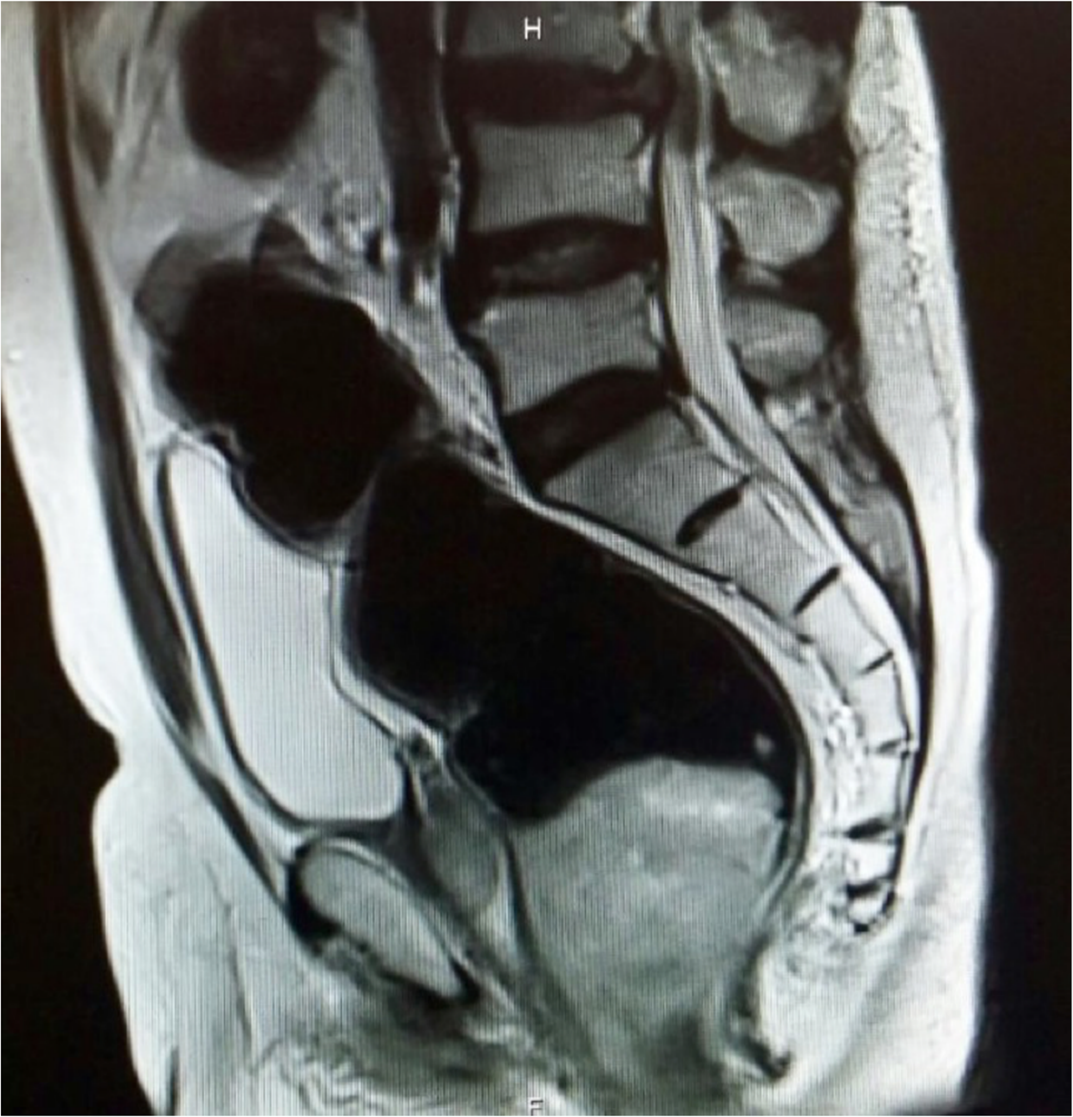
Sagittal T2-weighted magnetic resonance image shows hyperintense areas of the distal rectum mass, showing no invasion to bladder or prostate

The investigation proceeded with a colonoscopy that showed a 5 cm elevated lesion covered by normal mucosa, located 4 cm above the pectineal line. Complementary pelvic magnetic resonance imaging revealed a well-defined round tumor arising from his distal rectum associated with mild colon distension (Figs. 1 and 2). There were no signs of prostatic or bladder invasion. His urine analysis was within normal limits.

**Fig. 3:**
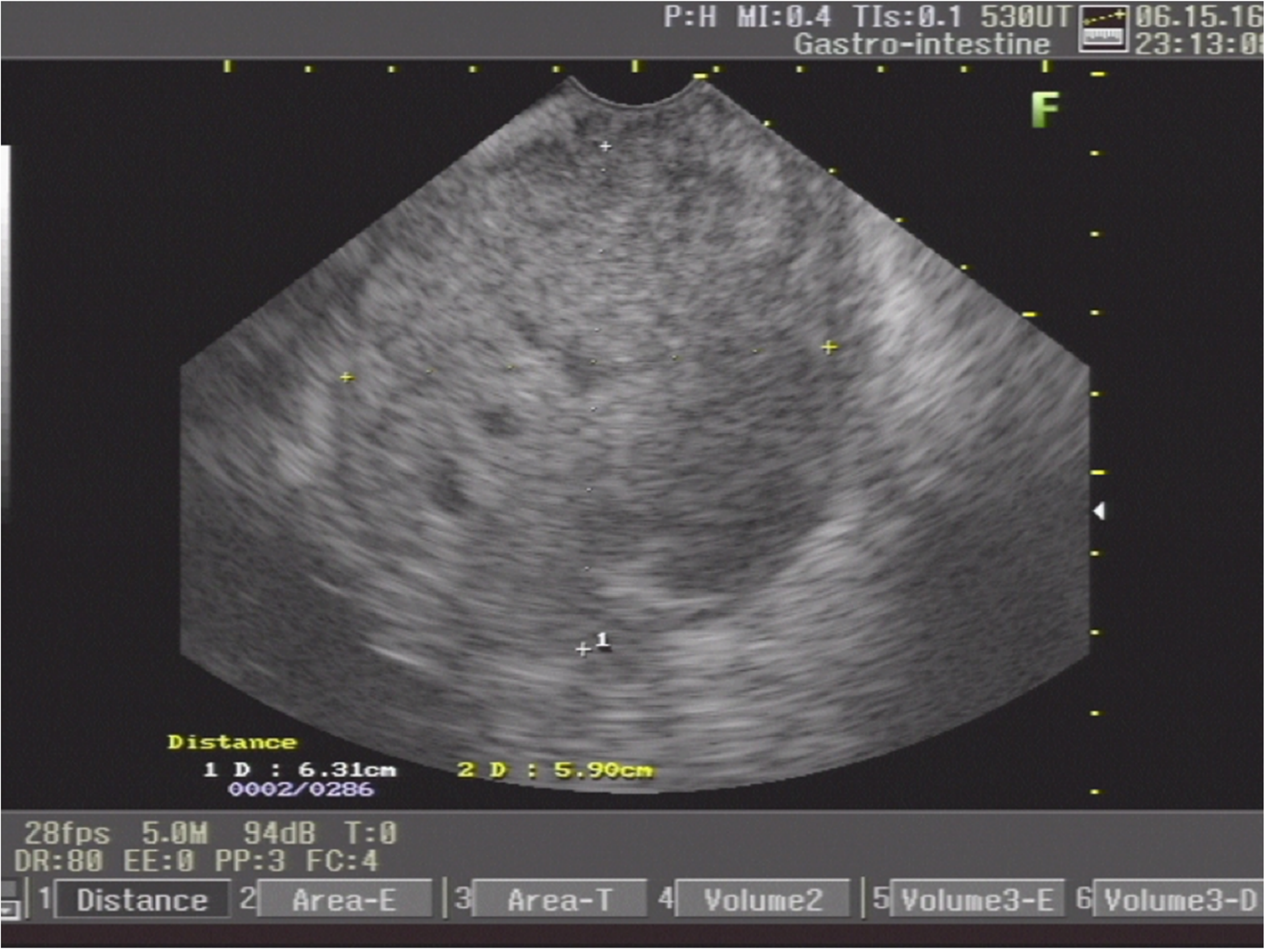
Endoscopic ultrasound showing a homogeneous hypoechoic mass with areas of necrosis as a rectal subepithelial lesion originating at the fourth layer (muscularis propria)

**Fig. 4:**
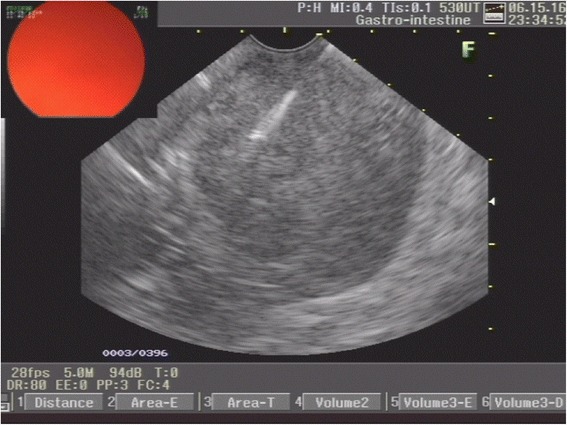
Endoscopic ultrasound-guided fine needle aspiration of the subepithelial lesion originating at the fourth layer (muscularis propria)

**Fig. 5:**
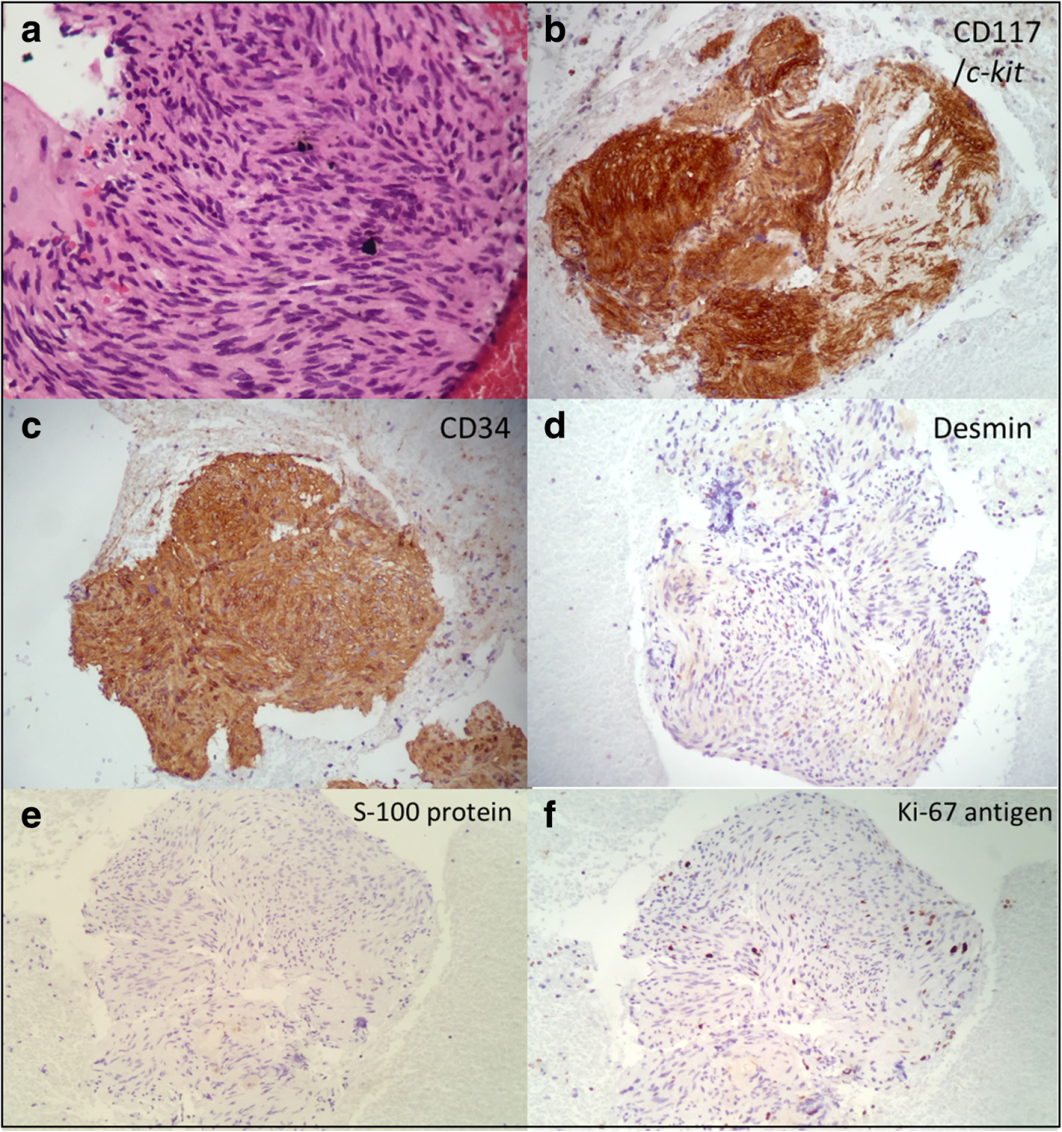
Rectal stromal tumor, immunohistochemistry immunostains. **a** Cell block showing syncytial tissue fragment with mixed spindle and epithelioid cells. Hematoxylin and eosin. **b** A strong positive staining with CD117/c-kit. **c** Strong positive reaction with CD34. **d** Negative reaction with desmin. **e** Negative reaction with S-100 protein. **f** Weak nuclear staining for Ki-67 in tumor cells

An EUS was then performed and confirmed the diagnosis of a homogeneous hypoechoic mass with areas of necrosis as a rectal subepithelial lesion originating at the fourth layer (muscularis propria; Fig. 3). EUS-guided fine needle aspiration (FNA) of the lesion was performed (Fig. 4), followed by cytological and immunohistochemistry evaluation, showing spindle and epithelioid cells of variable sizes, in fascicles separated by stroma. The cell borders were well defined, and their nucleus varied from oval to spindle-shaped with smooth membranes. The chromatin was coarsely granular and their cytoplasm was scant. The cell block preparation was extremely helpful in this case, particularly for immunostains, which confirmed the diagnosis of rectal GIST. The cells reacted strongly and consistently to CD117/c-kit and CD34, and negative for desmin and S-100 protein. There was weak staining for nuclear Ki-67 in tumor cells (Fig. 5). After a multidisciplinary meeting, an abdominoperineal resection of his rectum was decided. He underwent surgery; pathology of the specimen confirmed the diagnosis of rectal GIST. He is now asymptomatic after 3 months’ follow-up and is on adjuvant therapy with tyrosine-kinase inhibitor determined by our local Oncology group.

## Discussion

GISTs are mesenchymal tumors that display differentiation toward the lineage of interstitial cells of Cajal [7] and typically express CD117/c-kit. Recent studies showed that these cells play a fundamental role in gastrointestinal motility because they conduct inputs from enteric motor neurons, generating intrinsic electrical rhythmicity in phasic smooth muscles [8]. On histological examination, the cells of Cajal are at the fourth layer (muscularis propria) [8, 9]. For that reason, GISTs present as SETs.

A definitive diagnosis of a GIST tumor depends on the association of histological and immunohistochemical findings. On histological examination, there are three types of GIST: spindle cell type (70%), epithelioid (25%), and mixed subtype (5%) [10]. The most sensitive and specific immunohistochemistry marker is CD117/c-kit while other standard markers are platelet-derived growth factor receptor-alpha (PDGFR-α) and CD34 [7]. Nonetheless, recent studies showed that a new marker, Discovered On GIST-1 (DOG-1), plays a significant role in diagnostic yield of GIST tumors, especially when CD117 expression has not been demonstrated. Therefore, DOG1 has recently been recommended to be part of the routine diagnostic panel [11].

GISTs are equally distributed between genders, with a mean age of presentation of 60 to 65-years old (ranging from 10 to 100 years) [12]. Although GISTs are a rare type of cancer, they are the most common sarcoma of the gastrointestinal tract [1] with an estimated incidence of 1/100,000 per year [13]. Treatment is responsible for an enormous financial impact; the estimated cost per month per patient is approximately US$4000 for non-recurrent cases and more than US$8000 for recurrent cases [14].

Approximately 80% of patients are symptomatic, despite the non-specificity of symptoms (mild abdominal pain, gastrointestinal bleeding, and fatigue due to iron deficiency anemia). The most common primary affected site is the stomach while esophagus, colon, and rectum are the rarest [12]. GISTs account for only 0.1% of cases of rectal tumors [15].

Characteristics identified clinically and on esophagogastroduodenoscopy (EGD) are well known to help differentiate between GISTs and non-GIST SETs. Patients of an older age, tumor site location outside the gastric cardia, large tumor size, exophytic growth pattern, and ulceration or dimpling are independent preoperative predictive factors for GISTs versus non-GISTs [16].

Metastatic progression of GISTs it is not uncommon. It is expected that almost 30% of all completely resected GISTs will recur within 2 years [17]. Most of the recurrences are disseminated, especially involving the liver and peritoneum [18]. Patients presenting a metastatic GIST are treated mainly with imatinib (tyrosine-kinase inhibitor) therapy and the median overall survival rate is 5 years [19]. In this context, positron emission tomography (PET) with ^18^F-fluorodeoxyglucose (^18^F-FDG) tracer may provide valuable information. A recent systematic review showed that ^18^F-FDG PET was a strong predictor of clinical outcome and in assessing treatment response [20]. Other studies found that it provides a timelier and more accurate response assessment compared to a computed tomography (CT) scan and magnetic resonance imaging (MRI) [21]. Moreover, ^18^F-FDG PET may provide information about the malignancy: a tumor presenting maximum standardized uptake value (SUV) greater than 3.0 has a high malignant potential even in small tumors (<2 cm) [22]. Therefore, this imaging modality should be considered in all cases of GISTs, especially in metastatic disease, and while monitoring treatment response.

Regarding EUS evaluation, GISTs are seen as fourth layer tumors. The main differential diagnoses are leiomyoma, leiomyosarcoma, and schwannoma [23]. Precise diagnosis is essential because each of the pathologies mentioned above has a different treatment, follow-up, and prognosis. For small leiomyomas (<2 cm), surveillance annually with EGD may be performed [24, 25]. If the lesion is larger, symptomatic, or growing, then surgical resection is advised. Small leiomyomas (<2 cm) may be treated with endoscopic resection instead of surgical resection. The endoscopic resection of leiomyomas has lesser perforation rates than GISTs resection [26]. Leiomyosarcomas have a worse prognosis compared to GISTs, as their recurrence rate and subsequent metastasis rate may reach 70% and 80% respectively [27]. Schwannomas are benign tumors that tend to recur locally and to become malignant. Their response to chemotherapy and radiotherapy remains uncertain [28]; consequently, the treatment of choice for schwannomas is surgical resection with free margin. If they are uniformly benign, the long-term outcome is excellent [29]. Furthermore, the standard treatment for GISTs is open or laparoscopic surgical resection with free margins [30], although recent studies demonstrated the feasibility and safety of endoscopic resection through an endoscopic submucosal dissection (ESD) technique for small tumors. GISTs have been associated with a relatively low recurrence rate during long-term follow-up despite little R_0_ resection rate [31]. Other prospective multicenter studies are needed to assess the real role of endoscopy in the treatment of GISTs, but initial data are encouraging. Such differences among SETs enhance the need of a precise histological diagnosis. GISTs in a rectal location are related to worse outcomes compared to other sites [13]; this adds emphasis to the importance of precise and early diagnosis as in our reported case.

In this context, EUS is an important method that presents excellent diagnostic rates and may provide material for cytological and histological evaluation [4]. As previously described, GISTs are fourth layer tumors, which are hypoechoic and homogeneous. Differential diagnoses are leiomyoma and schwannoma [23]. Some echographic characteristics to differentiate GISTs from other fourth layer tumors have already been described in the literature. GISTs and schwannomas usually exhibit a complete or incomplete marginal hypoechoic halo, while leiomyomas do not show any clear marginal halo. Also, GIST echogenicities, in general, are low but slightly higher than that of the normal surrounding fourth muscle layer (muscularis propria), whereas the level of echogenicity in leiomyomas was nearly equal to that of the surrounding typical muscularis propria layer, and the echogenicity in schwannomas was extremely low. The difference in echogenicities among the fourth layer tumors (mesenchymal) might reflect the structural components and pathological differences of cellularity of these tumors [5].

Despite these characteristics, diagnostic accuracy for third and fourth layer tumors by EUS alone is around 50% [32]. A recent study proposed a scoring system for diagnosis of GISTs and other SETs based on EUS characteristics and found sensitivity and specificity to be 75% and 85%, respectively, for GISTs. However, this study only enrolled gastric SETs and had not been validated yet [33]. Therefore, a complementary biopsy is mandatory. Despite its high rates of inadequate material acquisition [6], FNA biopsy is still currently considered the procedure of choice for preoperative diagnosis of GISTs. The average diagnostic accuracy rate of EUS-guided FNA ranges from 60 to 80% in SETs [34]. Recent studies showed that Trucut biopsy is related to greater accuracy rates, and that should be preferred over FNA [35, 36]. Others studies propose dual biopsy (FNA and Trucut) to increase diagnostic yield [37]. However, there is no consensus yet, and hence FNA remains as the most performed procedure. To the best of our knowledge, no study assessed the sensitivity and specificity of the association of EUS features and Trucut or EUS-guided FNA.

Besides diagnostic yield, EUS also provides prognostic information: specific ultrasonography characteristics related to malignancy in GIST. Size larger than 20 mm, presence of cystic spaces, surface ulceration, irregular borders, and echogenic foci are unique features predictive of the malignant potential of GISTs and, therefore, worse outcomes [6, 38]. Among them, tumor size is the most important factor.

Other imaging methods may be useful for identification and to make the correct diagnosis of rectal GISTs. Because the rectum is an unusual location for a GIST, few studies assessed the role of MRI and a CT scan, but features such as large well-circumscribed masses and exophytic masses with moderate and heterogeneous enhancement on CT and MRI are associated with rectal GISTs. Furthermore, invasion of adjacent organs is uncommon, as well as regional lymph node enlargement, and small bowel obstruction [39]. If present, however, these must be identified and treated accordingly.

Finally, a combination of clinical, radiological, endoscopic, ultrasonographic, and cytological characteristics could eventually compose a scoring system, which would objectively enhance the preoperative diagnostic rates of GISTs and other SETs. Which would, therefore, help physicians to decide more accurately whether to, how to, and when to treat their patients.

## Conclusions

GISTs are rare tumors, and among their variety of primary locations the rectum is one of the rarest. This tumor location site has worse outcomes and higher morbidity rates. We report this rare case to emphasize the need for precise diagnosis and the important role of EUS-guided FNA in such situations. More studies and new technology are required to assess which is the best diagnostic approach for SETs and GISTs. For now, EUS-guided FNA is essential.

## Abbreviations

^18^F-FDG: ^18^F-fluorodeoxyglucose
bpm: Beats per minute
CT: Computed tomography
DOG1: Discovered on GIST-1
EGD: Esophagogastroduodenoscopy
ESD: Endoscopic submucosal dissection
EUS: Endoscopic ultrasound
FNA: Fine needle aspiration
GIST: Gastrointestinal stromal tumor
PDGFR-α: Platelet-derived growth factor receptor-alpha
PET: Positron emission tomography
SETs: Subepithelial tumors
SUV: Standardized uptake value
